# Effects of concurrent training on speed and agility performance in soccer referees

**DOI:** 10.3389/fphys.2026.1768715

**Published:** 2026-01-30

**Authors:** Barış Baydemir, Zülbiye Kaçay, Laurentiu-Gabriel Talaghir, Paula Ivan

**Affiliations:** 1 Faculty of Sports Sciences, Çanakkale Onsekiz Mart University, Çanakkale, Türkiye; 2 Faculty of Physical Education and Sport, Dunarea de Jos University of Galati, Galati, Romania

**Keywords:** agility, concurrent training, physical performance, soccer referees, speed

## Abstract

**Purpose:**

Soccer referees are exposed to high aerobic and anaerobic demands during match play, yet evidence regarding training strategies that simultaneously enhance speed and agility in this population remains limited. This study aimed to examine the effects of a 12-week concurrent training program on sprint and agility performance in soccer referees and to evaluate the sustainability of these effects through a follow-up assessment. To our knowledge, few intervention studies have simultaneously examined both sprint speed and agility performance in soccer referees and evaluated whether these adaptations are retained during a follow-up period. Importantly, the inclusion of a follow-up assessment provides evidence on the retention of training-induced adaptations, which has rarely been examined in referee populations under applied field-based training conditions.

**Methods:**

Fifty male soccer referees officiating in amateur leagues were assigned to a control group (n = 25) or an experimental group (n = 25). Both groups completed standard referee training twice weekly for 12 weeks, while the experimental group additionally performed concurrent training sessions combining endurance-based interval running and strength–power exercises twice per week. Sprint and agility performance were assessed using the 100 m sprint test and the Illinois Agility Test at pre-test, post-test, and 7-week follow-up. Data were analyzed using two-way mixed repeated measures ANOVA.

**Results:**

Significant Group × Time interaction effects were observed for both sprint and agility performance (p < 0.01) with moderate-to-large interaction effects. The experimental group demonstrated greater improvements in 100 m sprint and Illinois Agility Test performance compared with the control group following the intervention. Importantly, these performance gains were largely maintained at follow-up, indicating sustained training adaptations.

**Conclusion:**

A 12-week concurrent training program integrated into standard referee conditioning resulted in significant and sustained improvements in speed and agility performance. These findings highlight the effectiveness of concurrent training as a multidimensional approach to enhancing physical capacities that are critical for soccer refereeing and support its practical implementation within referee training programs. These results support the integration of concurrent training into referee conditioning programs to improve movement efficiency and match positioning capacity. Practitioners may consider concurrent training as a feasible strategy to improve and maintain key physical qualities required for match positioning across competitive phases.

## 1 Introduction

Football is a sport characterized by its high tempo and constantly changing dynamics, which require not only players but also referees to demonstrate superior physical and cognitive performance (Pancar et al., 2025). Referees, beyond enforcing the rules of the game, must keep up with the pace of play, maintain optimal positioning, and make accurate decisions in real time (Yousefian et al., 2023) Consequently, aerobic endurance, anaerobic capacity, speed, and agility are among the key physical performance components that determine refereeing performance (Castagna et al., 2007; Weston et al., 2019; Martin-Sanchez et al., 2022). Referees present a distinct movement profile compared with players. While players perform position-specific efforts interspersed with tactical pauses, referees must continuously adjust their speed and direction to maintain an optimal viewing angle and distance from the ball, often requiring repeated short accelerations, decelerations, and changes of direction across the full match duration. This continuous repositioning under time pressure makes sprinting speed and agility particularly relevant determinants of refereeing performance and decision-making quality (Zhang et al., 2025).The structural evolution of modern football and the increasing game tempo have made it essential for referees to perform at a physical level comparable to that of players. Research has shown that referees cover between 10 and 13 km during a match, with a large proportion consisting of high-intensity activities such as sprints, accelerations, and rapid changes of direction (Castagna et al., 2007; Castagna et al., 2019). Despite the recognized importance of speed and agility for referee positioning and match control, these performance components have received considerably less attention in training-based intervention studies compared to aerobic endurance. Although repeated sprint ability and acceleration are also relevant to refereeing, sprint speed and change-of-direction ability are directly linked to rapid repositioning during transitions of play and maintaining optimal viewing angles. Therefore, these outcomes were prioritized as they represent practical determinants of positioning quality during match-critical situations. Within this context, speed and agility are critical for maintaining appropriate positioning, reacting quickly to dynamic situations, and supporting accurate decision-making, while endurance is fundamental for sustaining both physical and cognitive performance throughout the match (MacMahon et al., 2007).

Given the combined aerobic and anaerobic demands placed on soccer referees, training approaches that simultaneously target multiple physical performance components are required. As the level of competition increases, the physical demands placed on referees also rise. This necessitates not only improved aerobic capacity but also development in sprint speed, agility, and strength key anaerobic performance parameters. In recent years, various training strategies aimed at improving referees’ physical capacities have become a focus of sports science research. Among these strategies, concurrent training defined as the systematic combination of endurance and strength training has emerged as an effective method to support multidimensional performance improvements (Yousefian et al., 2023; Zhang et al., 2025). In the present study, concurrent training refers specifically to the planned combination of endurance and strength–power training stimuli within the same training program (and within the same session), which differs from ‘hybrid’ or ‘mixed’ training terms that are sometimes used more broadly without a structured sequencing rationale. Traditional training approaches may be insufficient to meet the multifaceted physical demands of refereeing, highlighting the need for more integrated and comprehensive training methods (Demir, 2015; Baydemir et al., 2021). While concurrent training has been widely investigated in athletic populations, its specific effects on key anaerobic performance indicators in soccer referees remain largely unexplored.

Concurrent training aims to simultaneously improve endurance, speed, agility, and strength components, thereby contributing to overall physical performance. However, studies specifically examining the effects of concurrent training on the speed and agility performance of soccer referees remain scarce. Given the high aerobic and anaerobic demands placed on referees, they represent a unique and meaningful population for exploring this training approach (Prieto-González and Sedlacek, 2022).

Weston et al. (2009) demonstrated that the physical exertion of referees during matches is largely comparable to that of players, with elite referees in the Premier League successfully matching game tempo. The accuracy of referees’ decision-making is closely linked to their physical fitness, as it enables correct positioning a concrete indicator of performance. This relationship becomes more pronounced at higher levels of competition (Castillo et al., 2016). FIFA’s physical fitness standards further outline the capacities required for referees to officiate at the international level (FIFA, 2008).

In recent years, a variety of training models have been developed and applied to enhance referees’ physical performance. Within this context, concurrent training has gained attention as a multifaceted preparation strategy, enabling the simultaneous development of endurance and strength capacities. However, a notable gap remains in the literature concerning its effects on key anaerobic performance indicators such as sprinting speed and agility among referees. Investigating these effects is crucial both scientifically and practically, as improvements in these capacities may enhance referees’ movement efficiency, reaction speed, and in-game decision-making (Baydemir et al., 2020; Fadde and Zaichkowsky, 2018).

To date, there is no clear consensus regarding training models that effectively enhance both aerobic and anaerobic performance components in soccer referees. Despite the growing interest in referee performance optimization, no consensus has been reached regarding the most effective training models to simultaneously target both aerobic and anaerobic capacities in this population. This lack of evidence represents a critical gap in the current literature.

Therefore, the present study aimed to investigate not only the immediate effects but also the sustainability of a 12-week concurrent training program on speed and agility performance in soccer referees. It was hypothesized that referees undertaking concurrent training would demonstrate greater and more sustained improvements in sprint and agility performance compared to those performing standard referee training alone. Although concurrent training is generally expected to improve multiple fitness qualities, the novelty of the present study lies in (i) targeting two referee-relevant anaerobic outcomes (sprint speed and agility) rather than predominantly aerobic endpoints, and (ii) including a follow-up assessment to evaluate the retention of adaptations, which is rarely addressed in intervention studies in soccer referees. Findings from this study are expected not only to enhance the physical preparedness of referees but also to contribute to improving the overall quality, tempo, and fluidity of the game through better positioning and decision-making during matches.

## 2 Methods

### 2.1 Participants

A total of 50 male soccer referees (25 control, 25 experimental) officiating in amateur in the Tekirdağ region of Turkey participated voluntarily in this study. Participants had a minimum of 3 years of officiating experience and were actively officiating at least one match per week during the competitive season. *A priori* power analysis was conducted using G*Power 3.1 (α = 0.05, power = 0.80, effect size f = 0.25), indicating that a minimum of 44 participants were required. The effect size assumption (f = 0.25) was based on a moderate Group × Time interaction expected for sprint and agility performance outcomes, consistent with previous concurrent training interventions. To account for potential dropouts, 50 participants were recruited. Participants were randomly assigned to either the experimental or control group using a computer-generated randomization sequence.

The study protocol was conducted in accordance with the principles of the Declaration of Helsinki. Written informed consent was obtained from all participants prior to participation. Ethical approval was granted by the Institutional Ethics Committee of Çanakkale Onsekiz Mart University, Turkey (Approval No: 2025–48).

### 2.2 Research model/experimental design

The study employed a randomized controlled pre-test, post-test, and follow-up design over a 21-week period. Both groups performed standard referee training twice weekly for 12 weeks (Tuesdays and Thursdays), consisting of activities such as endurance runs, sprint drills, and game-related movement patterns (45 ± 5 min per session). In addition, the experimental group completed concurrent training twice per week (Wednesdays and Fridays), designed to combine endurance and resistance components within the same session.

The concurrent training protocol consisted of:

Endurance training: 20–25 min of interval running (4 × 4 min at 85%–90% HRmax, 3 min active recovery between intervals) (Helgerud et al., 2007). Strength/power training: 20 min including bodyweight and plyometric exercises (e.g., squats, lunges, bounding, horizontal jumps) and resisted sprints (Loturco et al., 2017). Intensity was progressively increased every 3 weeks.

The experimental group performed two additional training sessions per week; therefore, the total weekly training frequency and volume were higher than in the control group. This reflects a practical applied model but should be considered when interpreting group differences.

The control group continued with standard referee training only. Testing sessions were performed at three time points: pre-test (Week 1), post-test (Week 14), and follow-up (Week 21). The follow-up assessment was included to evaluate the sustainability of training-induced performance adaptations. All training sessions were supervised by certified strength and conditioning specialists to ensure proper execution and intensity control.

### 2.3 Training program and testing schedule

The intervention lasted 21 weeks and consisted of a 12-week concurrent training program followed by a 7-week maintenance period during which only standard referee training was continued. Training was scheduled four times per week, with referee training (RT) conducted on Tuesdays and Thursdays and concurrent training (CT) on Wednesdays and Fridays. Each session lasted approximately 45 ± 5 min. The control group performed only RT sessions, whereas the experimental group performed both RT and CT.

Baseline testing was conducted in Week 1 over three non-consecutive days to minimize fatigue effects: anthropometric measurements on Tuesday, 100 m sprint testing on Wednesday, and Illinois Agility testing on Friday. Post-intervention testing (Week 14) followed the same schedule. Follow-up testing (Week 21) included only the sprint and agility tests, conducted on Tuesday and Thursday, respectively. All testing took place at the same outdoor track facility under standardized environmental conditions (temperature 20 °C–24 °C, no precipitation).

Table 1 summarizes the training implementation and testing timeline.

**TABLE 1:** Training implementation and study timeline.

Week/Day	Tuesday	Wednesday	Thursday	Friday
Week 1	Pre-test (anthropometric)	Pre-test (100 m Sprint)	Rest	Pre-test (Illinois agility)
Weeks 2–13	Referee training (RT)	Concurrent training (CT)	Referee training (RT)	Concurrent training (CT)
Week 14	Post-test (anthropometric)	Post-test (100 m Sprint)	Rest	Post-test (Illinois agility)
Weeks 15–20	RT	Rest	RT	Rest
Week 21	Follow-up (100 m Sprint)	Rest	Follow-up (Illinois agility)	Rest

RT = referee training; CT = concurrent training. Baseline (Week 1) and post-intervention (Week 14) testing were conducted over three non-consecutive days, while follow-up testing (Week 21) included only sprint and agility assessments. All training sessions lasted approximately 45 ± 5 min.

#### 2.3.1 Anthropometric measurements

Standing height and body weight were measured in the morning under fasting conditions using a Dikomsan BW 200 stadiometer and scale. Body mass index (BMI) was calculated using the standard formula: BMI = weight (kg)/height^2^ (m)^2^ (Benazeera, 2014).

#### 2.3.2 Performance tests

##### 2.3.2.1 100 m sprint test

Sprint performance was assessed on a standard athletics track using a telemechanique photoelectric timing system positioned at the start and finish lines. After a standardized warm-up, participants started from a standing position 0.5 m behind the start line. Each participant performed three maximal sprints over 100 m with 3-min rest intervals between trials. The fastest time was recorded (Kamnardsiri et al., 2024). The 100 m sprint test was selected to assess maximal sprinting speed and speed endurance, which are relevant for repeated high-intensity running demands experienced by referees during match play.

#### 2.3.3 Illinois Agility Test

Agility performance was assessed using the Illinois Agility Test as described by Raya et al. (2013). The course measured 10 m in length and 5 m in width, with three cones placed at 3.3 m intervals down the center. Timing gates (Sevilen Electronics SE-160) were placed at the start and finish lines. After familiarization trials and a standardized warm-up, participants performed two maximal trials from a prone start position with hands touching the ground. The best time (s) was recorded, with 2-min rest intervals between attempts. The Illinois Agility Test was selected because it includes multiple directional changes and acceleration–deceleration demands and has been used in referee and field-sport fitness testing contexts. While other COD tests may better mimic specific refereeing patterns, the Illinois test provides a standardized and reliable measure for evaluating agility changes following training.

### 2.4 Statistical analysis

Data were analyzed using IBM SPSS Statistics 29. Missing data and outliers were examined using boxplots and z-scores; none were detected. Descriptive statistics were calculated for age, height, weight, and BMI. The normality of distribution was assessed using the Shapiro–Wilk test due to the sample size (n < 30 per group) (Field, 2024).

Baseline equivalence between groups was verified using independent-samples t-tests for pre-test sprint and agility scores. A two-way mixed repeated measures ANOVA (Group × Time) was used to examine changes in performance measures over time. The assumption of sphericity was assessed using Mauchly’s test, and Greenhouse–Geisser corrections were applied when violations were detected. Post-hoc pairwise comparisons were performed using Bonferroni-adjusted tests. Partial eta squared (η^2^) was calculated as a measure of effect size and interpreted according to Cohen’s guidelines (small = 0.01, medium = 0.06, large = 0.14). For pairwise comparisons, Cohen’s d effect sizes (with 95% confidence intervals) were calculated to provide a practical interpretation of within- and between-group differences. Effect sizes were reported to provide a magnitude-based interpretation of the observed effects. Statistical significance was set at p < 0.05.

## 3 Results

A total of 50 participants completed the study, with no dropouts reported. All variables were normally distributed according to the Shapiro–Wilk test (p > 0.05). Independent-samples t-tests revealed no significant differences between the experimental and control groups at baseline for age, height, body mass, BMI, sprint performance, or agility performance (p > 0.05). Overall, after the intervention, the experimental group demonstrated greater improvements in both 100 m sprint and Illinois Agility Test performance compared with the control group. Importantly, these performance gains remained largely intact at follow-up, demonstrating the durability of the training adaptations.

Table 2 presents the baseline characteristics of the participants. No significant differences were observed between the experimental and control groups for age, height, body mass, or BMI (p > 0.05), indicating that the groups were comparable prior to the intervention.

**TABLE 2:** Baseline characteristics of the participants.

Variable	Group	n	Mean ± SD	Range
Age	Control	25	22.24 ± 2.10	14–25
	Experimental	25	22.40 ± 1.90	14–25
Height (m)	Control	25	1.80 ± 0.06	1.70–1.94
	Experimental	25	1.79 ± 0.05	1.70–1.94
Body Mass (kg)	Control	25	74.11 ± 5.4	65–85
	Experimental	25	75.13 ± 5.8	65–85
BMI (kg/m^2^)	Control	25	2.70 ± 1.8	19.07–26.64
	Experimental	25	23.21 ± 2.0	19.07–26.64

Values are presented as mean ± standard deviation (SD). BMI, body mass index. No significant differences were observed between groups at baseline (p > 0.05).

Table 3 presents the baseline (pre-test) comparisons between the experimental and control groups for 100 m sprint and Illinois Agility performance. Independent-samples t-tests showed no significant differences between the groups for either sprint or agility variables (p > 0.05), indicating that both groups were comparable prior to the intervention.

**TABLE 3:** Baseline (pre-test) comparisons between groups.

Variable	Measurement	Group	n	Mean	SD	t (df)	p-value
100 m Sprint (s)	Pre-test	Experimental	25	13.66	0.24	0.04 (48)	0.966
		Control	25	13.66	0.23		
Illinois agility (s)	Pre-test	Experimental	25	21.18	1.38	−1.25 (48)	0.218
		Control	25	21.62	1.10		

Values are presented as mean ± standard deviation (SD). Independent-samples t-tests indicated no significant baseline differences between groups for sprint or agility performance (p > 0.05).

Table 4 presents 100 m sprint performance across pre-test, post-test, and follow-up measurements for the experimental and control groups.

**TABLE 4:** 100 m sprint performance across testing sessions.

Group	Pre-test (mean ± SD)	Post-test (mean ± SD)	Follow-up (mean ± SD)	n
Control	13.66 ± 0.24	13.67 ± 0.25	13.76 ± 0.22	25
Experimental	13.66 ± 0.23	13.57 ± 0.23	13.58 ± 0.24	25

Values are presented as mean ± standard deviation (SD) and expressed in seconds (s).

Table 5 summarizes the results of the two-way mixed repeated measures ANOVA conducted on 100 m sprint times. The analysis revealed a significant Group × Time interaction (F (2,48) = 7.11, p = 0.001, partial η^2^ = 0.13), indicating that the experimental group improved significantly more than the control group over time. Neither the main effect of Time (F (2,48) = 2.65, p = 0.076, partial η^2^ = 0.05) nor Group (F (1,48) = 2.55, p = 0.117, partial η^2^ = 0.05) reached statistical significance. Post hoc comparisons showed that significant differences occurred between post-test and follow-up within the experimental group, whereas no significant changes were found in the control group. The experimental group improved by 0.09 s from pre- to post-test and retained this improvement at follow-up (−0.08 s vs. pre-test), whereas the control group showed no change at post-test and a slight decline at follow-up (+0.10 s vs. pre-test).

**TABLE 5:** Two-way mixed repeated measures ANOVA for 100 m sprint.

Effect	F	df	p-value	Partial η^2^	Significant pairwise differences
Time (pre, post, follow)	2.65	(2, 48)	0.076	0.05	—
Group (experimental vs. control)	2.55	(1, 48)	0.117	0.05	—
Group × time	7.11	(2, 48)	0.001	0.13	Post vs. follow (experimental)

Results of the two-way mixed repeated measures ANOVA, for 100 m sprint performance. Partial eta squared (η^2^) values represent effect sizes.

**FIGURE 1:**
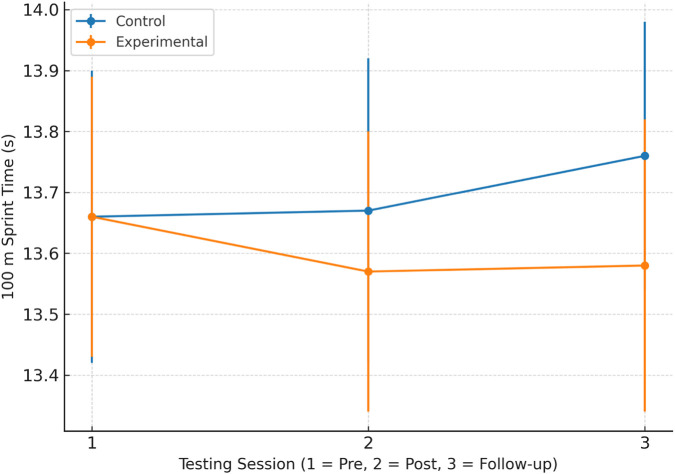
100 m sprint performance across testing sessions for the experimental and control groups. *Values represent mean ± SD for pre-test (1), post-test (2), and follow-up (3*). 100 m sprint performance across testing sessions for the experimental and control groups. Values are presented as mean ± standard deviation (SD) for pre-test (1), post-test (2), and follow-up (3).

Figure 1 illustrates these time-dependent changes in sprint performance for both groups.

A two-way mixed repeated measures ANOVA was conducted to examine sprint performance across time and between groups (Table 5). The main effect of Time was not statistically significant (F (2,48) = 2.646, p = 0.076), and the main effect of Group was also not significant (F (1,48) = 2.551, p = 0.117). A significant Group × Time interaction was observed (F (2,48) = 7.106, p = 0.001, partial η^2^ = 0.129). Post hoc comparisons identified a significant difference between post-test and follow-up measurements within the experimental group.

**FIGURE 2:**
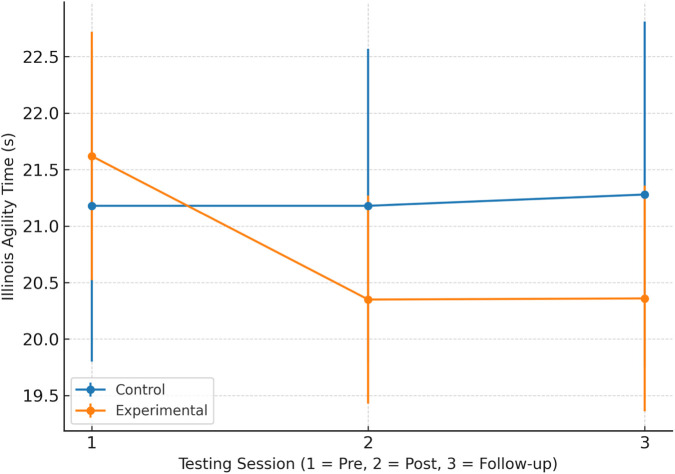
Illinois Agility Test performance across testing sessions for the experimental and control groups. Values are presented as mean ± standard deviation (SD) for pre-test (1), post-test (2), and follow-up (3).

Table 6 presents the Illinois Agility Test performance across pre-test, post-test, and follow-up sessions for both groups. Figure 2 illustrates the Illinois Agility Test performance across pre-test, post-test, and follow-up sessions for both groups.

**TABLE 6:** Illinois agility test performance across testing sessions.

Group	Pre-test (mean ± SD)	Post-test (mean ± SD)	Follow-up (mean ± SD)	n
Control	21.18 ± 1.38	21.18 ± 1.39	21.28 ± 1.53	25
Experimental	21.62 ± 1.10	20.35 ± 0.92	20.36 ± 1.00	25

Values are presented as mean ± standard deviation (SD) and expressed in seconds (s).

Repeated measures ANOVA was conducted to examine changes in agility performance across time and between groups (Table 7). A significant main effect of Time was observed (F (2,48) = 32.11, p < 0.001, partial η^2^ = 0.401), whereas the main effect of Group was not significant (F (1,48) = 1.68, p = 0.201). A significant Group × Time interaction was found (F (2,96) = 37.41, p < 0.001, partial η^2^ = 0.443). Post hoc comparisons identified significant differences between pre-test and both post-test and follow-up measurements within the experimental group. The experimental group improved by 1.27 s from pre- to post-test and maintained this improvement at follow-up (−1.26 s vs. pre-test), whereas the control group showed no change at post-test and a small decline at follow-up (+0.10 s vs. pre-test).

**TABLE 7:** Two-way mixed repeated measures ANOVA for Illinois agility performance.

Effect	F	df	p-value	Partial η^2^	Significant pairwise differences
Time (pre, post, follow)	32.11	(2, 48)	<0.001	0.40	1–2, 1–3
Group (experimental vs. control)	1.68	(1, 48)	0.201	0.03	—
Group × time	37.41	(2, 96)	< 0.001	0.44	1–2, 1–3 (experimental)

Results of the two-way mixed repeated measures ANOVA, for Illinois Agility Test performance. Partial eta squared (η^2^) values represent effect sizes.

## 4 Discussion

This study examined the effects of a 12-week concurrent training program on speed and agility performance in soccer referees. The main findings indicate that concurrent training elicited statistically significant changes in sprint and agility performance over time, with differential responses observed between the experimental and control groups. Importantly, performance changes observed following the intervention were partially maintained during the follow-up period, suggesting the presence of residual training adaptations relevant to refereeing performance. To contextualize these findings, the results can be compared with concurrent training evidence in soccer players and related field-sport populations. Specifically, sprint time improved by ∼0.09 s and agility time by ∼1.27 s after the intervention, with these gains largely retained at follow-up.

These findings are consistent with player-based concurrent training studies showing that combining strength–power and endurance training can improve sprint and change-of-direction performance when program structure and recovery are appropriately managed. The present data extend this evidence to soccer referees, a population with distinct movement and decision-making demands. Sanchez-Garcia et al. (2018) reported that sprint performance in soccer referees is closely related to endurance capacity, while Romano et al. (2021) emphasized the effectiveness of the Illinois Agility Test in evaluating referee fitness. In this context, the present findings extend existing evidence by demonstrating that agility performance in referees can be positively influenced through an integrated training approach combining endurance and strength-related stimuli. Beyond prior observational evidence in referees, the present intervention provides applied support for integrating endurance and strength–power stimuli within a structured weekly schedule.

Previous studies have suggested that training programs for referees should emphasize high-intensity exercise and anaerobic threshold speed to enhance aerobic capacity (Castagna et al., 2019). The concurrent training model applied in the present study incorporated both aerobic and anaerobic components, aligning with these recommendations. The integration of endurance-based interval training with strength and plyometric exercises may have contributed to improvements in neuromuscular coordination, rate of force development, and running economy, which are critical determinants of sprinting and change-of-direction performance in referees. In addition, Casajus and Castagna (2007) demonstrated that aerobic fitness supports sustained performance in referees across different age groups, highlighting the importance of individualized and multidimensional training strategies. However, these physiological mechanisms were inferred from performance changes and were not directly measured; therefore, they should be interpreted cautiously. Although the present study focused on physical outcomes, these adaptations may also be relevant for cognitive and perceptual aspects of refereeing performance.

Beyond physical performance, moderate-to-high intensity exercise has been shown to positively influence cognitive functions relevant to refereeing (Senécal et al., 2021). Although cognitive performance was not directly assessed in the present study, improvements in physical capacities such as speed and agility may indirectly support decision-making by facilitating optimal positioning and reducing fatigue during match play. Given the limited number of intervention studies in referees, additional insights can be drawn from concurrent training research in comparable populations, while acknowledging sport-specific differences.

While limited research has specifically examined concurrent training in soccer referees, evidence from other populations supports the present findings. Prieto-González and Sedlacek (2022) reported that concurrent training effectively improves both strength and endurance, whereas Huiberts et al. (2024) highlighted that training adaptations may vary depending on individual characteristics such as sex and training background. These findings underscore the importance of considering individual differences when designing training programs for referees. A key consideration in concurrent training is the potential “interference effect,” which may influence power-related adaptations depending on program design.

Meta-analytic evidence suggests that concurrent training does not impair maximal strength but may influence explosive power development depending on program structure (Schumann et al., 2022). Given the importance of rapid acceleration and directional changes in refereeing, careful sequencing and load management of endurance and strength components appear essential. Furthermore, the use of running-based endurance modalities combined with bodyweight and plyometric exercises in the present study may have helped balance aerobic adaptations without excessive interference with speed-related performance (Lundberg et al., 2022). Although concurrent training may induce an interference effect under certain conditions, the present program included strength–power exercises and running intervals within a structured weekly schedule, which may have minimized interference. Moreover, progressive overload and supervision likely ensured adequate intensity distribution and technical quality.

From a broader perspective, concurrent training has been associated with systemic adaptations beyond physical fitness, including improvements in executive cognitive functions and aerobic capacity in different populations (Li et al., 2024; Jimeno-Almazán et al., 2022). Together, these findings support the notion that concurrent training represents a multidimensional training approach capable of eliciting comprehensive physiological adaptations.

In conclusion, the present findings highlight that soccer referees require structured and well-planned training approaches that target not only endurance but also anaerobic performance components such as speed and agility. The concurrent training model appears to be an effective and practical method to enhance the physical capacities required for modern soccer refereeing. Accordingly, integrating concurrent training principles into referee conditioning programs may help better match the multidimensional demands of match officiating. Future studies should examine the effects of concurrent training across different age groups, genders, competitive levels, and training volumes, as well as explore its potential influence on match-related performance indicators. From a practical standpoint, adding two concurrent training sessions per week to standard referee training may be a feasible strategy to enhance sprint and agility capabilities that support match positioning. Coaches and referee fitness instructors may consider integrating interval-based endurance work with plyometric and resisted sprint drills, while ensuring adequate recovery and progressive overload. Importantly, the maintenance of performance gains at follow-up suggests that adaptations may persist even when the concurrent stimulus is removed, although ongoing exposure to some high-intensity elements may be required for long-term retention.

## 5 Conclusion

This study demonstrated that a 12-week concurrent training program was associated with statistically significant and partially sustained improvements in both sprint and agility performance among soccer referees. Performance changes observed across pre-, post-, and follow-up measurements suggest that concurrent training can elicit both immediate and residual adaptations relevant to the physical demands of soccer refereeing.

These findings underline the importance of incorporating training strategies that simultaneously target aerobic and anaerobic performance components in referee development programs. Accordingly, concurrent training may represent a practical approach to enhancing the multidimensional physical fitness required for modern soccer refereeing.

From an applied perspective, integrating concurrent training protocols into regular referee conditioning may support physical capacities relevant to match-related performance demands. Future studies should specifically examine female referees and elite-level officials, and evaluate different training volumes, durations, and modalities to optimize concurrent training prescriptions across competitive levels.

## 6 Limitations

Several limitations of the present study should be acknowledged. First, the study sample consisted only of male soccer referees, which limits the generalizability of the findings to female referees. Second, participants were recruited from a single regional context, which may restrict the external validity of the results across different competitive environments. Third, although improvements in physical performance were observed, match-related performance indicators and cognitive outcomes were not directly assessed. In addition, match-performance indicators (e.g., GPS-derived high-speed running, acceleration counts, and positioning metrics) were not collected. Moreover, physiological mechanisms underlying performance improvements (e.g., neuromuscular adaptations or running economy) were inferred and not directly measured. Finally, the 100 m sprint test was used as a global measure of maximal sprint performance; while informative, shorter sprint distances or repeated sprint tests may provide more sport-specific insights for refereeing performance. Additionally, the experimental group completed a higher weekly training frequency and total volume than the control group, which reflects an applied model but may have contributed to the observed group differences.

## Data Availability

The raw data supporting the conclusions of this article will be made available by the authors, without undue reservation.
